# Persistent T cell-mediated immune responses against Omicron variants after the third COVID-19 mRNA vaccine dose

**DOI:** 10.3389/fimmu.2023.1099246

**Published:** 2023-01-23

**Authors:** Milja Belik, Oona Liedes, Saimi Vara, Anu Haveri, Sakari Pöysti, Pekka Kolehmainen, Sari Maljanen, Moona Huttunen, Arttu Reinholm, Rickard Lundberg, Marika Skön, Pamela Österlund, Merit Melin, Arno Hänninen, Antti Hurme, Lauri Ivaska, Paula A. Tähtinen, Johanna Lempainen, Laura Kakkola, Pinja Jalkanen, Ilkka Julkunen

**Affiliations:** ^1^ Institute of Biomedicine, University of Turku, Turku, Finland; ^2^ Department of Health Security, Finnish Institute for Health and Welfare, Helsinki, Finland; ^3^ Clinical Microbiology, Turku University Hospital, Turku, Finland; ^4^ Department of Infectious Diseases, Turku University Hospital, Turku, Finland; ^5^ Department of Internal Medicine, Lapland Central Hospital, Rovaniemi, Finland; ^6^ Department of Paediatrics and Adolescent Medicine, Turku University Hospital and University of Turku, Turku, Finland

**Keywords:** COVID-19, mRNA vaccines, T cell responses, third vaccine dose, booster vaccine, omicron

## Abstract

**Introduction:**

The prime-boost COVID-19 mRNA vaccination strategy has proven to be effective against severe COVID-19 disease and death. However, concerns have been raised due to decreasing neutralizing antibody levels after COVID-19 vaccination and due to the emergence of new immuno-evasive SARS-CoV-2 variants that may require additional booster vaccinations.

**Methods:**

In this study, we analyzed the humoral and cell-mediated immune responses against the Omicron BA.1 and BA.2 subvariants in Finnish healthcare workers (HCWs) vaccinated with three doses of COVID-19 mRNA vaccines. We used enzyme immunoassay and microneutralization test to analyze the levels of SARS-CoV-2 specific IgG antibodies in the sera of the vaccinees and the in vitro neutralization capacity of the sera. Activation induced marker assay together with flow cytometry and extracellular cytokine analysis was used to determine responses in SARS-CoV-2 spike protein stimulated PBMCs.

**Results:**

Here we show that within the HCWs, the third mRNA vaccine dose recalls both humoral and T cell-mediated immune responses and induces high levels of neutralizing antibodies against Omicron BA.1 and BA.2 variants. Three weeks after the third vaccine dose, SARS-CoV-2 wild type spike protein-specific CD4^+^ and CD8^+^ T cells are observed in 82% and 71% of HCWs, respectively, and the T cells cross-recognize both Omicron BA.1 and BA.2 spike peptides. Although the levels of neutralizing antibodies against Omicron BA.1 and BA.2 decline 2.5 to 3.8-fold three months after the third dose, memory CD4^+^ T cell responses are maintained for at least eight months post the second dose and three months post the third vaccine dose.

**Discussion:**

We show that after the administration of the third mRNA vaccine dose the levels of both humoral and cell-mediated immune responses are effectively activated, and the levels of the spike-specific antibodies are further elevated compared to the levels after the second vaccine dose. Even though at three months after the third vaccine dose antibody levels in sera decrease at a similar rate as after the second vaccine dose, the levels of spike-specific CD4^+^ and CD8^+^ T cells remain relatively stable. Additionally, the T cells retain efficiency in cross-recognizing spike protein peptide pools derived from Omicron BA.1 and BA.2 subvariants. Altogether our results suggest durable cellmediated immunity and protection against SARS-CoV-2.

## 1 Introduction

SARS-CoV-2 has been circulating globally since its emergence at the end of 2019. To date, different variants of concerns (VOCs) have caused multiple waves of infections, leading to hundreds of millions of COVID-19 cases with more than 6 million deaths. Recent studies demonstrate that SARS-CoV-2 infection and used vaccines induce a relatively strong immunological response including the formation of memory B cells, neutralizing antibodies, and T cell-mediated immune responses (1–4). Neutralizing antibodies have been considered a good correlate of protection (5). However, the constantly emerging new variants and the waning antibody levels (6) predispose the population to recurrent infections.

During spring 2022, Omicron subvariants BA.1 and BA.2 were the dominant VOCs in Europe and Finland, and in June 2022 the newly emerged Omicron subvariants BA.4 and BA.5 became the most prevalent variants circulating in the population. Significant concerns are raised for the new Omicron sublineages since BA.4/5 variants seem to be effective in evading the pre-existing vaccine or disease-induced immunity (7, 8). To compete with declining antibody levels and the constantly evolving SARS-CoV-2 variants, a third dose was recommended in the European Union starting in the fall of 2021, and a fourth vaccine dose has been recommended for people older than 60 years of age or at risk starting in the fall of 2022. Recurrent immunizations have been shown to boost the neutralizing antibody levels (9), however, more knowledge on the efficacy and duration of the T cell responses towards VOCs is crucially needed for decisions concerning the timing and composition of additional COVID-19 vaccine doses.

Here we have analyzed humoral and cell-mediated immune responses against Omicron BA.1 and BA.2 subvariants in Finnish health care workers (HCWs) vaccinated with three doses of mRNA vaccines. We show that the third vaccine dose induces high and well-retained T cell responses and high, but declining neutralizing antibody titers against Omicron variants. Importantly, after the third vaccine dose, the vaccinees present equally good memory T cell responses toward the wild type and BA.1 and BA.2 variant spike peptides.

## 2 Materials and methods

### 2.1 Study population

**Figure 1:**
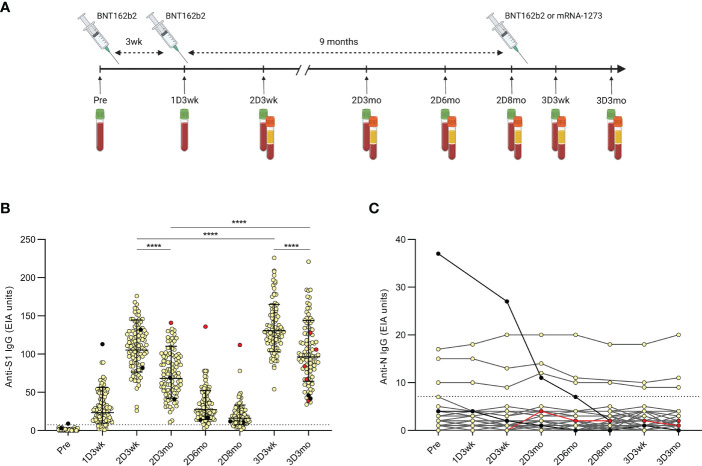
Timeline of the COVID-19 vaccinations and samplings of the HCWs and antibody levels after the second and third COVID-19 mRNA vaccine doses. **(A)**, Serum samples and peripheral blood mononuclear cells (PBMCs) were collected at regular intervals from health care workers (HCWs, n=100) vaccinated with two sequential doses of BNT162b2 (BioNTech-Pfizer) and a third mRNA vaccine dose of either BNT162b2 (n=46) or mRNA-1273 (Moderna; n=54). Sera were collected before the first vaccination and up to three months after the third dose and PBMCs were collected from a proportion of the HCWs (n=32) at indicated time points. **(B)**, SARS-CoV-2 S1-specific IgG antibody levels were measured with EIA from the serum samples collected before the first vaccination (Pre; n=100), three weeks after the first vaccination (1D3wk; n=99), three weeks (2D3wk; n=100), three months (2D3mo; n=99), six months (2D6mo; n=100), and eight months (2D8mo; n=85) after the second vaccine dose, and three weeks (3D3wk; n=100) and three months (3D3mo; n=94) after the third vaccine dose. Geometric means with geometric SDs are shown. Wilcoxon signed-rank test was used to analyze the statistical significance between time points (2D3wk vs 2D3mo; 2D3wk vs 3D3wk; 3D3wk vs 3D3mo; 2D3mo vs 3D3mo), and two-tailed p-values <0.05 were considered statistically significant. ****p<0.0001. **(C)**, SARS-CoV-2 N-specific IgG antibody levels were measured with EIA from the same samples as the S1-specific IgG antibodies. Black dots represent HCWs with a PCR-confirmed SARS-CoV-2 infection prior to the first vaccination (n=2) and red dots represent HCWs with a breakthrough infection after the second vaccine dose (n=1) or after the third vaccine dose (n=4). Cut-off values are indicated with dashed lines.

The study population consisted of three times vaccinated Turku University Hospital health care workers (HCWs, n=100) randomly selected from a larger cohort (10, 11) vaccinated with two sequential doses of BNT162b2 mRNA COVID-19 vaccine (BioNTech-Pfizer) in a three-week interval and with the third dose of either one of the two mRNA vaccines: BNT162b2 (n=46) or mRNA-1273 (Moderna; n=54). Sera were collected from the vaccinees before the first vaccination and in regular intervals up to three months after the third vaccine dose (**Figure 1A**). At the same study visit, peripheral blood mononuclear cells (PBMCs) were collected from 32 HCWs at indicated time points (**Figure 1A**). Samples three weeks after the third vaccine dose were collected between October 2021 and January 2022, and samples three months after the third dose were collected between December 2021 and March 2022. At every sample collection, the participants filled out a symptom questionnaire and in case of being symptomatic, they were encouraged to take a COVID-19 RT-qPCR test arranged as part of a local infection control practice. The study was carried out under the ethical permission of the Southwest Finland health district (ETMK 19/1801/2020, EudraCT 2021-004419-14). All participants were assigned written informed consent before the collection of the first study sample.

### 2.2 SARS-CoV-2 S1 and N protein antibody enzyme immunoassay

An in-house EIA was used to analyze the levels of SARS-CoV-2 spike protein subunit (S1) and nucleoprotein (N) specific IgG antibodies in the sera of vaccinees as described previously (10). Briefly, 96-well plates were coated with purified recombinant SARS-CoV-2 antigens (3.5 µg/ml of S1 and 2.0 µg/ml of N). Serum samples were diluted to 1:300 (N-based EIA) and 1:1000 (S1-based EIA), and the amount of bound IgG was quantified with an absorbance measurement at a 450nm wavelength. Linear interpolation between the optical density (OD) values of the known positive (100 EIA units) and negative control (0 EIA units) serum samples was used to convert the OD values to EIA units. The cut-off values for seropositivity were as described previously (12).

### 2.3 SARS-CoV-2 variants

The SARS-CoV-2 isolates in this study were FIN25-20 (Pango lineage B.1, D614G strain, GenBank ID MW717675.1 and GISAID ID EPI_ISL_412971), FIN1-20 (lineage B, MZ934691 and EPI_ISL_407079), FIN37-21 (lineage B.1.617.2, Delta variant, OK626882.1 and EPI_ISL_2557176), FIN55-21 (lineage B.1.1.529.1, Omicron BA.1 variant, EPI_ISL_8768822.2), FIN58-22 (lineage B.1.1.529.2, Omicron BA.2 variant, OP199045 and EPI_ISL_9695067) and FIN61-22 (lineage B.1.1.529.5, Omicron BA.5 variant, OP199047 and EPI_ISL_13118918). Viruses were isolated from SARS-CoV-2 PCR-positive nasopharyngeal samples in VeroE6 (for FIN25-20 and FIN20-20) or VeroE6-TMPRSS2-H10 cells (13) (for FIN37-21, FIN55-21, FIN58-22 and FIN61-22) and further passaged in VeroE6-TMPRSS2-H10 cells in DMEM (FIN25-20, FIN37-21, FIN55-21 and FIN58-22) or in VeroE6 cells in EMEM (FIN20-20 and FIN61-22) supplemented with 2% FBS, 2mM L-glutamine, and penicillin-streptomycin. Median Tissue Culture Infectious Dose (TCID_50_) assay was used to determine the titer of the virus stocks. Amino acid changes in the SARS-CoV-2 isolates are indicated in the **Supplementary Table 1**.

### 2.4 Microneutralization test

A microneutralization test (MNT) was used to measure the *in vitro* neutralization capacity of the sera, as described previously (10, 14). MNTs were performed in two laboratories (A and B). Briefly, for each serum sample, two-fold dilution series was prepared in duplicates on a 96-well plate starting from 1:10 dilution into 50µl of DMEM (laboratory A) or EMEM (laboratory B) supplemented with 2% fetal bovine serum (FBS), 2mM L-glutamine, and penicillin-streptomycin. The virus was added to the serum dilutions in a concentration of 50 (laboratory A) or 100 (laboratory B) TCID_50_ resulting in 1:20 final serum dilution and incubated for 1h at +37°C. VeroE6-TMPRSS2-H10 (laboratory A) or VeroE6 (laboratory B) cells were added (50 000 cells per well) into the virus-serum dilution mixture, bringing the final volume to 150µl. The final mixture was incubated at +37°C and 5% CO_2_ for four days. The cells were fixed with 4% formaldehyde (laboratory A) or 30% formalin (laboratory B), stained with crystal violet, and visualized for cell death. The neutralization titer was determined as the reciprocal of the serum dilution that was able to inhibit 50% of cell death. Serum dilution inhibiting 50% of cell death at a dilution of 1:20 or above was considered positive for neutralizing antibodies. Each plate included a serum with a known neutralizing antibody level as a positive control.

### 2.5 Activation induced marker assay

PBMCs were isolated and cryopreserved as previously described (1). PBMCs were thawed rapidly at +37°C and washed twice with RPMI-1640 culture medium (Gibco) supplemented with 10% human AB serum, 2mM L-glutamine, and penicillin/streptomycin. Cells were counted with TC20 automated cell counter, and ~1x10 (6) live cells per well were seeded in 96-well U-bottom plates (Thermo). PBMCs were stimulated with SARS-CoV-2 wild type and Omicron BA.1 and BA.2 variant spike protein peptide pools (PM-WCPV-S-1, PM-SARS2-SMUT08-1, and PM-SARS2-SMUT09-1, PepMiX, JPT Peptide Technologies) at a concentration of 0.5µg/ml. The full-length SARS-CoV-2 spike protein peptide pools were combined from two sub-pools consisting of 158 or 157 15-mer peptides (a few 13-17-mers) with 11 amino-acid overlaps. Omicron BA.1 and BA.2 peptide pools contained 38 and 31 aa changes, respectively, of which 21 were shared between the variants (**Supplementary Table 1**). Purified tetanus toxoid (10µg/ml, AJ Vaccines) was used as a positive control and DMSO (0.4%) was used as a negative control. Cells were incubated for 48h at +37°C and 5% CO2.

### 2.6 Flow cytometry analysis

After stimulation, PBMCs were washed with PBS supplemented with 0.01% NaN_3_ and stained with Zombie Green dye (BioLegend) at room temperature in the dark for 15min. PBMCs were washed with FACS buffer (PBS containing 2% fetal bovine serum and 0.01% NaN_3_) and incubated for 30min at +4°C in the dark with fluorochrome-labeled anti-human antibody mixtures recognizing CD45, CD3, CD4, CD8, CD69, CD134, and CD137, CD45RA, CCR7 and CXCR5 (**Supplementary Table 2**). PBMCs were washed with FACS buffer and suspended in 200µl of PBS supplemented with 0,01% NaN_3_. T cell subtypes were characterized with NovoCyte Quanteon Flow Cytometer (Agilent Technologies Inc) and analyzed with NovoExpress v1.5.0 (Agilent Technologies Inc). Gating of the cells was done manually from the main cell population of DMSO-stimulated samples, and the same gating was subsequently used for each respective tetanus- or S-peptide pool-stimulated cells. In case of a deviant CD4^+^ response to the positive control, the sample was excluded from the analysis. A stimulation index (SI) was calculated by dividing the percentage of AIM^+^ cells after SARS-CoV-2 spike peptide pool stimulation by the percentage of AIM^+^ cells after DMSO stimulation. If the percentage of AIM+ cells after stimulation was 0, the stimulation index was marked as the smallest value of the participant. The cut-off values for positive spike-specific CD4^+^ and CD8^+^ T cell responses were 2.5 and 1.25 SI, respectively, as described previously (1).

### 2.7 Cytokine detection with Luminex

Supernatants of the stimulated PBMCs were collected and measured for the levels of secreted cytokines IFN-γ, IL-2, and IL-4 with MILLIPLEXR Kit HCD8MAG-15K-03 (Millipore). Sample fluorescence was measured with Luminex MAGPIX magnetic bead analyzer (Luminex Corporation), and a median fluorescent intensity value was counted for seven diluted standards to calculate the concentration of each cytokine using a 5-parameter logistic regression. Only samples in the linear range were quantified. For statistical analyses samples below the lowest standard in the linear range were given half of the value of the standard (5 pg/ml for IFN-γ, 2 pg/ml for IL-2, and 10 pg/ml for IL-4). Samples over the highest standard in the linear range were given the highest value of the standard (5000 pg/ml for IFN-γ, 7500 pg/ml for IL-2, and 10 000 pg/ml for IL-4). Standard samples were done in duplicates and considered successful when the standard deviation was under 15%. According to the kit manufacturer, if there were less than 35 beads in the well, the samples could not be given a reliable concentration and thus those samples were discarded from the final analysis.

### 2.8 Statistical analysis

Data was collected in Excel 2016 (Microsoft 365) and analyzed in Prism v8 (GraphPad Software). Wilcoxon signed-rank test was used for paired samples when applicable, and the Kruskal-Wallis test, followed by Dunn’s multiple comparisons test, was used for comparisons when participants lacked samples from individual time points. Non-paired samples were tested with Mann-Whitney U-test. All tests were two-sided, and p-values <0.05 were considered statistically significant. Correlations were analyzed with Spearman’s correlation test. All statistical tests used in respective data analyses have been mentioned in the figure legends.

## 3 Results

### 3.1 SARS-CoV-2 S1 and N-specific antibody responses three months after the third vaccine dose

In this study, we continued the follow-up of a previously studied two-times BNT162b2 vaccinated HCWs (12, 15) who received one of the two available mRNA vaccines as the third dose (BNT162b2 n=46, mRNA-1273 n=54). Serum specimens were collected from before the first vaccination up to three months after the third vaccine dose (**Figure 1A**). Participants (n=100) were selected randomly, and they were 25–65 years old (mean 43 years, standard deviation (SD) 11 years) and 91% were female.

SARS-CoV-2 S1 and N specific IgG antibody levels were measured from all the participants with EIA as shown previously (12, 15). The second vaccine dose induced high levels of S1 specific antibodies, which declined in the subsequent months. The third vaccine dose effectively restored the amount of spike protein-specific antibodies, and the levels were even higher compared with the levels seen after the second dose (*p <*0.0001) (**Figure 1B**). Three months after the third vaccine dose, the kinetics of the decline in antibody levels was similar to the decrease observed after two vaccine doses (1.54-fold after the second vaccine dose and 1.35-fold after the third vaccine dose; *p <*0.0001).

We also observed a similar decline in the anti-S1 and anti-N IgG antibody levels of the vaccinees with a prior PCR-confirmed SARS-CoV-2 infection (n=2; black dots in **Figure 1B** and black dots and lines in **Figure 1C**). The decrease was likely due to the long period between infection and the first collected serum sample (30–78 days). One PCR-confirmed breakthrough infection was detected 47 days after the second vaccine dose (30 days before the sample collected three months after the second dose) with increased anti-S1 antibody levels (red dots in **Figure 1B**). However, the levels of anti-N IgG antibodies were only modestly elevated and stayed below the cut-off (red dots and line in **Figure 1C**). Four more PCR-confirmed breakthrough infections were identified 35–92 days after the third dose (21–83 days before the sample collected three months after the third dose), indicating a relatively low rate of breakthrough infections among HCWs during the study period. The vaccinees with steadily detectable low level anti-N IgG antibodies and with no known previous SARS-CoV-2 infection (n=3, **Figure 1C**) are likely due to cross-reactive antibodies from previous seasonal coronavirus infections (15).

### 3.2 Neutralizing antibodies against delta and omicron BA.1 and BA.2 variants after the third vaccine dose

**Figure 2:**
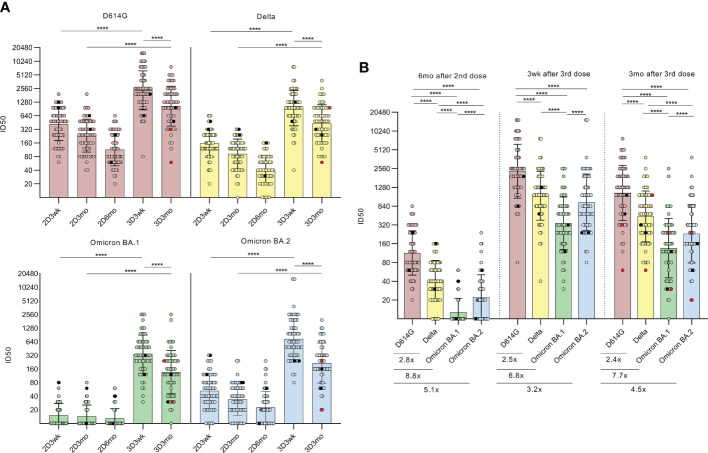
Neutralizing antibodies after the second and third COVID-19 mRNA vaccine doses. **(A)**, Microneutralization test was used to analyze the neutralizing antibody responses of HCWs (n=64) against D614G strain and variants Delta, BA.1, BA.2 after the second vaccine dose at three-week (2D3wk, n=60, BA.2 n=59), three-month (2D3mo, n=60, BA.2 n=59) and six-month (2D6mo, n=62, BA.2 n=61) time points as well as after the third vaccine dose at three-week (3D3wk, n=63, BA.2 n=62) and three-month (3D3mo, n=59, BA.2 n=58) time points. HCWs with PCR-confirmed SARS-CoV-2 infection prior to the first vaccination (n=2) are marked with black dots and breakthrough infections after the third dose (n=2) are marked with red dots. Differences between time points (2D3wk vs 3D3wk; 2D3mo vs 3D3mo; 3D3wk vs 3D3mo) were analyzed with Wilcoxon signed-rank test. **(B)**, Neutralization titers between D614G strain and variants Delta, BA.1, and BA.2 at six months after the second vaccine dose as well as three weeks and three months after the third vaccine dose were compared and are represented as fold differences below the figures. Wilcoxon signed-rank test was used to analyze the statistical significance, and two-tailed p-values <0.05 were considered statistically significant. Samples with no data on both data points were excluded from the analyses. The geometric means with geometric SDs are shown in the figures. ****p<0.0001.

In addition to measuring the levels of the SARS-CoV-2-specific antibodies by EIA, we analyzed the ability of serially collected serum samples from 64 HCWs to *in vitro* neutralize the D614G strain and three variants of concern: Delta and two Omicron sublineages, BA.1 and BA.2 (laboratory A, **Figure 2**). Neutralization was tested from serum samples collected at three weeks, and three and six months after the second vaccine dose, and three weeks and three months after the third vaccine dose (**Figure 1A**). Of the 64 vaccinees, two individuals had a prior PCR-confirmed SARS-CoV-2 infection diagnosed 49–100 days before the first vaccine dose (black dots in **Figure 2**), and two additional participants had a breakthrough infection diagnosed 35–83 days after the third dose (red dots in Fig 2).

Three weeks after the second dose, 100% of the serum samples neutralized the D614G strain (60/60) and the Delta variant (60/60), while 37% (22/60) and 92% (54/59) of the samples neutralized the Omicron BA.1 and BA.2 variants, respectively (**Figure 2A**). Even though the antibody levels gradually declined, 100% (62/62) and 90% (56/62) had neutralizing antibodies against the D614G strain and Delta variant, respectively, at six months post the second dose. Interestingly, at the same time, only 23% (14/62) had neutralizing antibodies against the BA.1 variant, while 66% (40/61) had neutralizing antibodies against the BA.2 (**Figure 2B**). The titers of neutralizing antibodies against the virus variants were significantly lower compared to the D614G strain, as the fold-decreases were 2.8 (Delta), 8.8 (BA.1), and 5.1 (BA.2) (**Figure 2B**). In addition, the difference between the BA.1 and BA.2 variants was statistically highly significant (*p <*0.0001).

At three weeks after the third vaccine dose, all sera had significantly elevated neutralization titers against all virus variants compared with the levels observed after the second vaccine dose (all *p <*0.0001; **Figure 2A**). Again, three months after the third dose, the neutralizing antibody titers declined, although the titers remained at relatively high levels (geometric mean titer (GMT) 1043, 437, 136, and 231 against D614G, Delta, BA.1, and BA.2, respectively). In addition, only one serum sample failed to neutralize Omicron BA.1 variant. Furthermore, no significant differences were detected in neutralization titers between vaccinees who received BNT162b2 (n=33) or mRNA-1273 (n=31) as the third vaccine dose (**Supplementary Figure 1**).

A proportion of the samples collected three months after the third dose (n=22) were analyzed in a collaborative laboratory (laboratory B) for the neutralizing antibodies against the wild type (wt) (14) and Omicron BA.2 and BA.5 variants (**Supplementary Figure 2**). Compared with the wt, the neutralizing titers were 14.7-fold lower against the BA.2 variant and 18.9-fold lower against the BA.5 variant, indicating a distinct decrease in the neutralization capacity of the sera against both Omicron variants. All the serum samples (22/22) neutralized the wt, whereas 78% (16/22) of the sera neutralized the variants BA.2 or BA.5. The results differed substantially compared to the other laboratory, however, the neutralization tests were performed with different assay settings and the difference in the neutralization titers was similar to what has been described before (15).

### 3.3 CD4^+^ T cell responses against wild type and omicron BA.1 and BA.2 sublineages after the third vaccine dose

**Figure 3:**
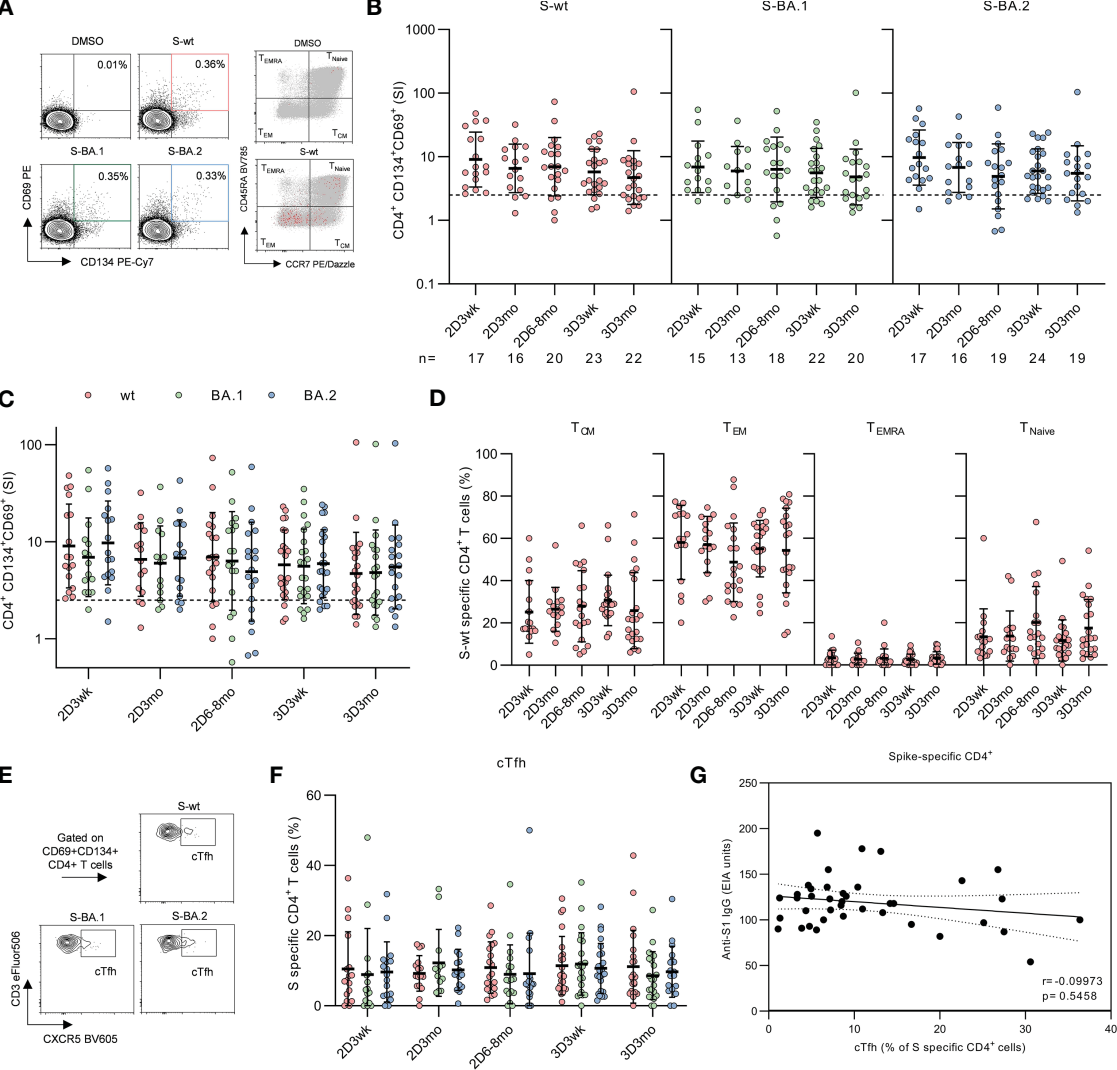
CD4^+^ T cell responses to SARS-CoV-2 wild type and Omicron BA.1 and BA.2 spike protein peptides after second and third mRNA vaccination doses. **(A)**, Representative flow cytometry plots and gating of CD4^+^ T cells expressing CD69^+^CD134^+^ and distribution of memory phenotypes of total CD4^+^ (gray) and activated CD4^+^CD69^+^CD134^+^ (red) T cells after stimulation with DMSO or SARS-CoV-2 (wt, BA.1, and BA.2) spike peptide pools. The gating strategy of CD4^+^ T cells is shown in **Supplementary Figure 3**. **(B)**, Longitudinal spike-specific CD4^+^ T cell responses were analyzed from 32 vaccinated HCWs three weeks (2D3wk), three months (2D3mo), and six to eight months (2D6–8mo) after the second vaccine dose, and three weeks (3D3wk) and three months (3D3mo) post the third vaccine dose. Data are shown as stimulation indices (SI) relative to DMSO-stimulated PBMCs. The dotted line indicates the cut-off SI and the numbers below represent the sample size. **(C)**, Comparison of SARS-CoV-2 wild type and Omicron BA.1 and BA.2 variant spike peptide pool specific CD4^+^ T cell responses. **(D)**, Proportions of memory phenotypes of SARS-CoV-2 wild type spike-specific CD4^+^ T cells after the second and third vaccine doses. Responses are shown only for samples with spike-specific CD4+ T cell response >1 SI. Total CD4^+^ T cell responses and distribution areshown in **Supplementary Figure 5**. **(E)**, Representative flow cytometry plots of circulating T follicular helper (cTfh, CXCR5^+^) cells expressing CD4^+^CD69^+^CD134^+^ in response to stimulation with SARS-CoV-2 wild type and Omicron BA.1 and BA.2 variant spike peptide pools. **(F)**, Proportion of CD4^+^CD69^+^CD134^+^ T cells expressing CXCR5^+^. **(G)**, Correlation between the SARS-CoV-2 S1-specific IgG antibody levels at three weeks after the second (n=16) and third (n=23) vaccine doses and SARS-CoV-2 wild type stimulated cTfh cells expressing CD4^+^CD69^+^CD134^+^ at corresponding time points. Error bars in panels b and c represent geometric means and geometric SDs, and in d and f means with SDs. In b and d, the statistical significance was determined with the Kruskal-Wallis test followed by Dunn’s multiple comparisons test since some participants were missing samples from individual time points. In c and f the statistical significance was determined with Wilcoxon signed-rank test for paired samples. CM; central memory, EM; effector memory, TEMRA; T effector memory CD45RA^+^.

For the analysis of CD4^+^ helper T cell-mediated vaccine responses, PBMCs were available from 32 HCWs at indicated time points after the second and third vaccine doses (**Figure 1A**). Some participants were missing individual time points. Since only six samples were collected eight months after the second dose (mean 7.8 months, SD 0.3 months) and 22 samples were collected six months after the second dose (mean 6.4 months, SD 0.4 months), the groups were combined into the same group (mean 6.7 months, SD 0.7 months; named as 2D6–8mo). The PBMCs were stimulated with tetanus toxoid and peptide pools covering the entire SARS-CoV-2 spike protein of the wild type (S-wt) and Omicron BA.1 and BA.2 variants, followed by the analysis of the expression of activation induced markers (AIM^+^, CD69^+^ and CD134^+^) by flow cytometry. Flow cytometry analysis was performed on 25/32 participants, while all the stimulated samples were included in cytokine analysis. The gating strategy is represented in **Figure 3A**, and **Supplementary Figures 3, 6A**. Antigen- specific CD4^+^ T cell responses were quantified as the frequencies of CD69^+^CD134^+^ cells relative to stimulation with a negative control stimulus (DMSO).

Two COVID-19 mRNA doses efficiently primed the spike-specific CD4^+^ T cells in all participants (17/17) and throughout the period post-primary vaccine series, CD4^+^ responses remained at clearly detectable levels, although the responses decreased slightly but non-significantly within eight months after the second dose (**Figure 3B**). Mean stimulation indices (SIs) for S-wt peptide pool stimulation were 9.0, 6.6, and 7.0 at three weeks, three months, and six to eight months after the second vaccine dose, respectively. The third vaccine dose did not increase the CD4^+^ responses, and the mean SI for S-wt stimulation was 5.8 at three weeks and 4.7 at three months post the third vaccine dose. Three weeks after the third dose, the response was observed in 87% (20/23) of the participants. CD4^+^ T cell responses against spike peptide pools of Omicron BA.1 and BA.2 were observed in 87% (13/15) and 94% (16/17) of the participants three weeks after the second dose and in 82% (18/22) and 83% (20/24) of the participants three weeks after the third dose, respectively. Despite a somewhat lower number of participants with CD4^+^ response against Omicron variant spike peptide pools compared to S-wt, the SIs were not significantly different between the wt and variant peptide pool stimulated cells (**Figure 3C**). In addition, tetanus toxoid specific responses remained unvaried at all time points (**Supplementary Figure 4B**).

Next, we analyzed the distribution of spike-specific CD4^+^ T cells into memory subsets by measuring the expression of CD45RA and CCR7, which allows the differentiation of central (T_CM_; CD45RA-CCR7^+^) and effector memory (T_EM_; CD45RA-CCR7-) cells from naive T cells (**Figure 3A**). After two mRNA vaccine doses spike-specific CD4^+^ responses were mainly composed of T_EM_ and T_CM_ phenotypes (**Figure 3D**). The percentages of T_EM_ and T_CM_ did not change substantially during the follow-up period or after the third vaccine dose, although a modest decrease of T_EM_ was observed at six to eight months post the second dose (11.4 percentage points compared to three weeks after the second dose). The distribution of CD4^+^ memory T cells did not differ after stimulation with S-wt and Omicron variant spike peptide pools (**Supplementary Figure 5A**).

In addition to analyzing the memory T cell subsets, we analyzed spike-specific circulating T follicular helper cells (cTfh) expressing the defining marker CXCR5^+^ (**Figures 3E–F**). Three weeks after the second dose, S-wt-specific cTfh cells were detected in 88% (15/17) of the participants and three weeks post the third vaccine dose cTfh cells were detected in 96% (22/23) of the participants. The percentages of spike-specific cTfh remained stable over the study period, and the responses were similar for all analyzed SARS-CoV-2 peptide pools. No correlation was observed between the number of S-wt-specific cTfh cells after the second dose and SARS-CoV-2 S1-specific antibodies against D614G after the third vaccine dose (Spearman r= -0.09973, p=0.5458, **Figure 3G**).

### 3.4 CD8^+^ T cell responses against wild type and Omicron BA.1 and BA.2 sublineages after the third vaccine dose

**Figure 4:**
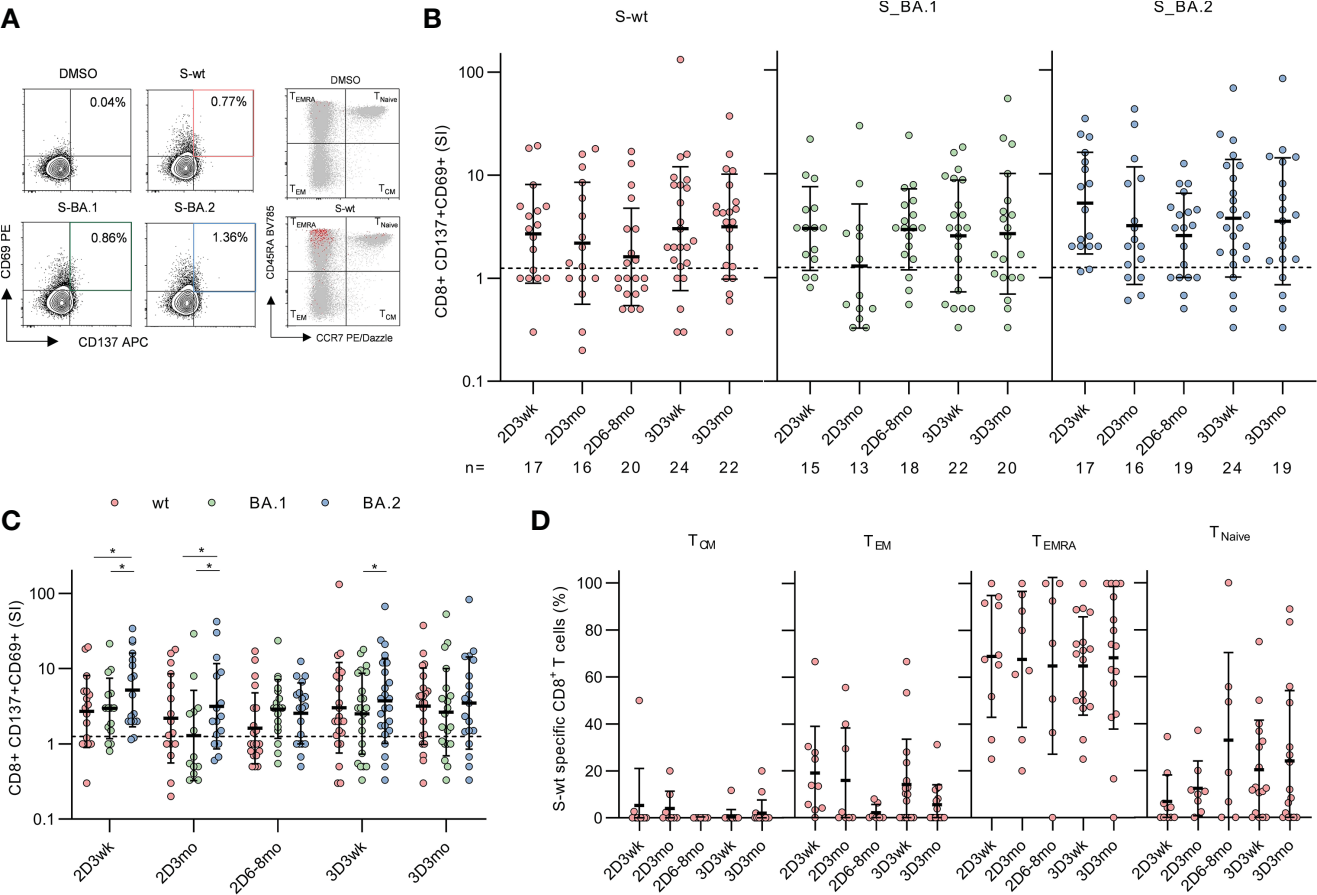
CD8^+^ T cell responses to SARS-CoV-2 wild type and Omicron BA.1 and BA.2 spike after the second and third mRNA vaccine dose. **(A)**, Representative flow cytometry plots and gating of CD8^+^CD69^+^CD137^+^ T cells and memory phenotypes of total CD8^+^ (gray) and activated CD8^+^CD69^+^CD137^+^ (red) T cells after stimulation with DMSO and SARS-CoV-2 (wt, BA.1, and BA.2) spike peptide pools. The gating strategy of CD8^+^ T cells is shown in **Supplementary Figure 3**. **(B)**, Spike-specific CD8^+^ T cell responses in 32 vaccinated HCWs three weeks (2D3wk), three months (2D3mo), and six to eight months (2D6–8mo) after two vaccine doses, and three weeks (3D3wk) and three months (3D3mo) post the third vaccine dose represented longitudinally. The dotted line indicates the cut-off SI and the numbers below represent the sample size. **(C)**, Cross-comparison of CD8^+^ T cell responses after stimulation with wild type and Omicron BA.1 and BA.2 spike peptide pools. **(D)**, Distribution of S-wt-specific CD8^+^ T cells expressing CD69^+^CD137^+^ into memory subsets after the second and the third vaccine dose. Responses are shown only for samples with spike-specific CD8+ T cell response >1 SI. Total CD8^+^ T cell responses and distribution are shown in **Supplementary Figure 5**. Error bars in **(B-C)** represent the geometric means with geometric SDs, and in d the means with SDs. In b and d, the statistical significance was determined with the Kruskal-Wallis test followed by Dunn’s multiple comparisons test since some participants were missing samples from individual time points. In **(C)**, the statistical significance was determined with Wilcoxon signed-rank test for paired samples: *p<0.05. CM, central memory; EM, effector memory; TEMRA, T effector memory CD45RA^+^.

In addition to CD4^+^ T cell responses, cytotoxic CD8^+^ T cell responses were analyzed in the AIM test. Spike-specific CD8^+^ T cells were identified by dual expression of activation markers CD69^+^ and CD137^+^ after tetanus toxoid and spike peptide pool stimulations. The gating strategy for activation and phenotypic markers is shown in **Figure 4A**, and **Supplementary Figures 3, 4A**.

S-wt-specific CD8^+^ T cell responses were generally lower than CD4^+^ responses, and three weeks after the second vaccine dose, a positive response was observed in 65% (11/17) of the participants (**Figure 4B**). Three and six months post the second vaccine dose, geometric mean SIs against S-wt peptide pool decreased to some extent (2.7 SI at three weeks, 2.2 SI at three months, and 1.6 SI at six to eight months after the second dose). This decline was, however, statistically not significant.

The third vaccine dose restored CD8^+^ responses to a higher level than the level after two vaccine doses (3.0 SI at three weeks after the third dose) (**Figure 4B**). Three weeks and three months after the third dose, 71% (17/24) and 77% (17/22) participants had a positive CD8^+^ response against the S-wt peptide pool, respectively. In addition, CD8^+^ T cells cross-recognized Omicron BA.1 and BA.2 spike peptide pools, although the responses between BA.1 and BA.2 spike protein peptide pools differed significantly at some time points (**Figure 4C**).

CD8^+^ T cells were mainly terminally differentiated effector memory cells expressing CD45RA^+^ (T_EMRA_) and a small proportion was T_EM_ cells (**Figure 4D**). The proportions of spike-specific T_EMRA_ cells remained constant during the study period, while the number of T_EM_ cells decreased to some extent at three months after the third vaccination. The frequency of CD8^+^ T cell subsets after stimulation with Omicron BA.1 or BA.2 spike peptide pools was similar to S-wt-stimulated cells (**Supplementary Figure 4B**). After stimulation with tetanus toxoid the CD8^+^ T cells were mainly T_EMRA_ and T_EM_ phenotypes (**Supplementary Figure 6D**).

### 3.5 The secretion of cytokines in SARS-CoV-2 spike peptide pool stimulated PBMCs and the correlations between secreted cytokines and spike-specific T cell responses

**Figure 5:**
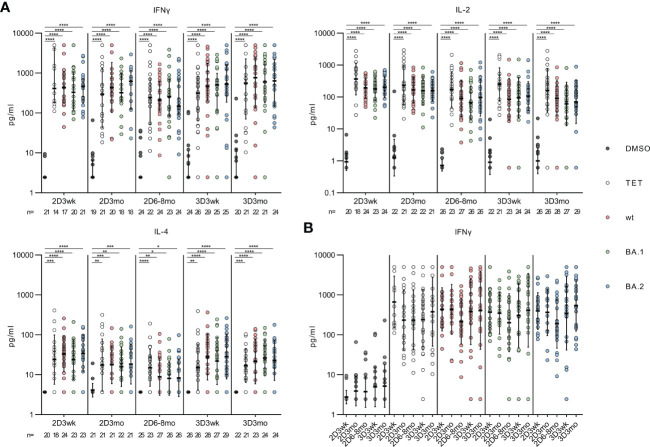
Effector cytokines IFN-γ, IL-2 and IL-4 secreted by the SARS-CoV-2 spike protein stimulated PBMCs. **(A)**, Secreted cytokines (IFN-γ, IL-2 and IL-4) were analyzed from the supernant of DMSO, tetanus toxoid (TET), and SARS-CoV-2 (wt, BA.1, and BA.2) spike protein peptide pool -stimulated PBMCs collected from 32 HCWs. **(B)**, Comparison of the secreted IFN-γ levels at indicated time points. The data are represented as geometric means with geometric SDs. The statistical significances were determined with the Kruskal-Wallis test followed by Dunn’s multiple comparisons test. *p<0.05; **p <0.01; ***p <0.001; ****p <0.0001.

In addition to flow cytometry analysis, we measured the supernatants of the stimulated PBMCs for the secretion of CD4^+^ and CD8^+^ T cell effector cytokines (IFN-γ, IL-2 and IL-4). At all the time points, the wt, Omicron BA.1 or Omicron BA.2 spike-specific peptide pool stimulations resulted in a statistically significant increase in the secretion of IFN-γ, IL-2, and IL-4 compared with the DMSO-control (all *p*<0.0001, **Figure 5A**). However, IL-4 was secreted in notably lower levels compared to other cytokines, and IL-4 levels declined slightly at three months and six to eight months after the second dose. The level of IFN-γ was also compared between the associated time points, and no statistically significant differences were seen within those (**Figure 5B**).

**Figure 6:**
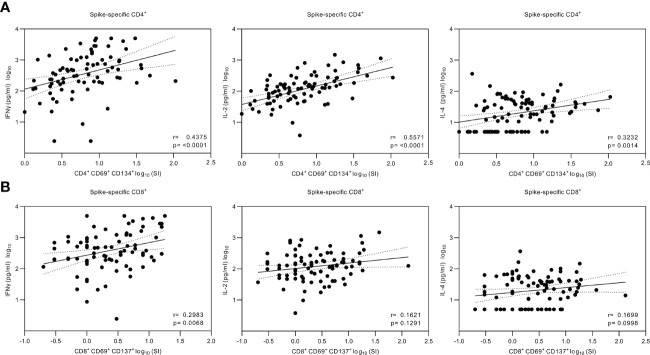
Correlation of the secreted cytokines and SARS-CoV-2 spike-specific T cell responses. **(A)**, Nonparametric Spearman correlation analysis of SARS-CoV-2 (wt) spike-specific CD4^+^ T cells and secreted IFN-γ, IL-2 or IL-4 cytokines measured from the supernatants of SARS-CoV-2 (wt) spike-stimulated PBMCs. The stimulated PBMCs were collected from 32 HCWs at three weeks, three, and six to eight months after the second vaccine dose and three weeks and three months after the third vaccine dose and grouped together for analysis (n=81 for IFN-γ, n=87 for IL-2, and n=88 for IL-4). **(B)**, Nonparametric Spearman correlation analysis of SARS-CoV-2 (wt) spike-specific CD8^+^ T cells and secreted cytokines (n=81 for IFN-γ, n=88 for IL-2, and n=88 for IL-4). Dotted lines indicate 95% confidence intervals (CI).

The secreted cytokine levels were analyzed for correlations with the CD4^+^ and CD8^+^ T cell responses (**Figure 6**). A statistically significant correlation was found between CD4^+^ T cell activation and the production of IFN-γ (r=0.438, *p*<0.0001) and IL-2 (r=0.557, *p*<0.0001), whereas the correlation between the CD4^+^ T cell activation and IL-4 production was weaker (r=0.323, *p*<0.01). The CD8^+^ cells had a higher correlation with the levels of secreted IFN-γ (r=0.298, *p<*0.01) compared with the levels of IL-2 (r=0.162, *p*>0.05) and IL-4 (r=0.170, *p*>0.05).

## 4 Discussion

In this study, we investigated the dynamics of the neutralizing antibody levels and memory CD4^+^ and CD8^+^ T cell responses after the third COVID-19 mRNA vaccine dose in Finnish HCWs. We demonstrate that after the administration of the third dose, both humoral and cell-mediated immune responses are effectively activated, and the levels of spike-specific antibodies are further elevated compared to the levels after the second vaccine dose. Three months after the third vaccine dose, antibody levels decrease at a similar rate as after the second vaccine dose, while spike-specific CD4^+^ and CD8^+^ T cells are relatively stable and effectively cross-recognize spike protein peptide pools from Omicron BA.1 and BA.2 variants. Our results support the findings of other T cell studies on mRNA-vaccinated individuals (3, 16, 17), indicating a durable cell-mediated immunity against SARS-CoV-2.

The recent emergence of Omicron subvariants, BA.1, BA.2, BA.4, and BA.5, have raised concerns on increased infection rates since two-times vaccinated have had an insufficient neutralizing antibody response against Omicron and antibody levels have decreased within months after the vaccination (18–20). In addition, real-world vaccine studies have demonstrated a decreased protective efficacy against BA.1 and BA.2 infections and six months after the second COVID-19 mRNA vaccine dose only a small proportion of vaccinees have remain protected against a symptomatic disease (<15%) (21–23). However, the third vaccine dose restored the efficacy to 56–74% against a symptomatic disease, suggesting the role of antibodies in preventing infections. Fortunately, protection against hospitalization has been high (>50%) despite the time point. There is also evidence that SARS-CoV-2 specific T cells protect from reinfections in the absence of antibodies in animal models (24, 25). Similar observations have been made in humans, where the severe disease was prevented even if the antibody levels were low or declined (26). Our results are well in line with these findings since even though we saw decreased neutralizing serum antibody levels against Omicron subvariants BA.1, BA.2, and BA.5, the vaccinees showed an efficient CD4^+^ and CD8^+^ T cell cross-recognition of Omicron spike peptides three months after the third vaccine dose.

As shown in previous studies, T cells recognize a broad range of linear epitopes which are not as sensitive to amino acid changes as epitopes on the surface of the proteins that are more specifically recognized by neutralizing antibodies (27). Mutated SARS-CoV-2 variant spike regions form only a small proportion of known CD4^+^ and CD8^+^ T cell epitopes (28, 29) and mutated epitopes do not significantly disrupt the overall T cell responses. Our previous study on two-times BNT162b2 vaccinated as well as studies done by others on COVID-19 mRNA vaccinated and convalescent individuals have not detected significant differences between T cell responses against spike protein peptides derived from ancestral SARS-CoV-2 strains or Alpha, Beta, Gamma, Delta, or Omicron BA.1 variants (2, 30–35) In the present study, we have extended the analysis to BA.2, and we show that despite the 31 aa changes in the BA.2 spike protein, the BA.2 spike protein peptide pool is cross-recognized by T cells as efficiently as the wt peptide pool.

In line with other studies (3, 22, 36, 37), SARS-CoV-2-specific memory CD4^+^ T cells induced by mRNA vaccines were mainly effector memory (T_EM_, CD4^+^CD45RA^-^CCR7^-^) and central memory (T_CM_, CD4^+^CD45RA^-^CCR7^+^) phenotypes, while the majority of SARS-CoV-2-specific CD8^+^ T cells were T_EM_ and CD45RA^+^ expressing effector memory (T_EMRA_, CD8^+^CD45RA^+^CCR7^-^) phenotypes irrespective of the variant spike peptides used in the analyses. On average, 25% and 58% of spike-specific CD4^+^ cells were T_CM_ and T_EM_, respectively, three weeks after the second vaccine dose and the third dose did not further increase the proportion of T_CM_ and T_EM_ cells. Effector cytokines secreted by the stimulated PBMCs represent Th1 type cytokine production pattern (increased IFN-γ and IL-2 production, and little IL-4 production). Th1 cells are known to stimulate the CD8^+^ T cell responses, and in line with this, the secretion of IFN-γ was associated with spike-specific CD8^+^ T cell responses.

We also detected the activation of SARS-CoV-2 spike-specific cTfh cells, which are considered to be critical for generating an efficient production of neutralizing antibodies. In line with one study (36), but in contrast to another study (22), the frequency of spike-specific cTfh cells remained unaltered for months after the second and third vaccine doses. Interestingly, we did not find a correlation between S1-specific or neutralizing antibodies and the number of activated cTfh cells, although other studies have shown a strong correlation between an early cTfh response and sequential antibody response (3, 22). This difference could be due to the experimental setting where other studies analyzed cTfh response after the first vaccine dose instead of after the second or third vaccine dose as was done in the present study. The data may indicate that cTfh responses are more important in primary antibody responses induced by the first vaccine dose. Altogether, the mRNA vaccines appear to be able to induce a highly coordinated and multi-functional cell-mediated immune response that likely correlates with protection against the severe form of COVID-19 disease.

One of the limitations of our study is a relatively small number of participants in flow cytometric analyses and missing data on different vaccine combinations (mRNA versus other types of vaccines) available in the EU and Finland. Secondly, in our T cell stimulation experiments, we used 15-mer pools of spike protein peptides that are suitable for efficient CD4^+^ cell stimulation but are not optimal for major histocompatibility complex class I (MHC-I) antigen presentation for low-frequency circulating CD8^+^ T cells that are better stimulated by smaller peptides. Thirdly, the more vulnerable elderly population, immunocompromised individuals, or children were not studied here and thus the study is skewed toward the healthy adult population. In addition, 91% of the study participants were female.

In conclusion, the third COVID-19 mRNA vaccine dose effectively restored and further increased humoral immune responses as well as maintained activated memory T cell responses. T cell responses remained detectable for at least seven months after the second and three months after the third vaccine dose. However, future studies are needed to analyze the long-term persistence of memory T cells. Based on our data, the benefit of further booster doses may be limited only to antibody responses in a corresponding population, and homologous vaccinations are unlikely to further expand the strength of T cell memory responses. Nevertheless, our data support the concept of T cell-mediated immunity being well conserved for relatively long periods after COVID-19 vaccination, and this may contribute to the protection against a severe disease even when neutralizing antibody levels decline. Our data provide relevant information for epidemiological data analysis and future vaccination strategies.

## Data availability statement

The raw data supporting the conclusions of this article will be made available by the authors, without undue reservation.

## Ethics statement

The studies involving human participants were reviewed and approved by Ethics Committee of Southwest Finland Health District. The patients/participants provided their written informed consent to participate in this study.

## Author contributions

MB, LK, PJ, and IJ designed the study. MB, OL, SV, AHa, SP, PK, LK, SM, MH, AR, and RL performed the experiments. MB, OL, SV, AHa, and PJ analyzed the data. LI, PT, and JL performed recruitment and sample and participant data collection. SP, AHu, and AHä contributed to the methodology and PÖ and MS provided key reagents. MM, JL, LK, PJ, and IJ supervised the study. MB, LK, PJ, and IJ wrote the manuscript. All authors contributed to the article and approved the submitted version.
