# Supplementary material for: Persistent T cell-mediated immune responses against Omicron variants after the third COVID-19 mRNA vaccine dose
Source: Front Immunol. 2023 Jan 23;14:1099246. doi: 10.3389/fimmu.2023.1099246 (PMC9899862; doi:10.3389/fimmu.2023.1099246)
Supplement: Supplementary file 1 [file DataSheet_1.docx]

**Supplementary Table 1.** Amino acid changes in the SARS-CoV-2 spike protein peptide pools and in the spike protein of SARS-CoV-2 isolates used in neutralization tests. Sequences of SARS-CoV-2 isolates are compared to Wuhan-Hu-1/2019 (MN908947). Amino acid changes in PepMiX peptide pools are obtained from JPT Peptides (https://www.jpt.com/clinical-indications/infections/sars-cov-2/).


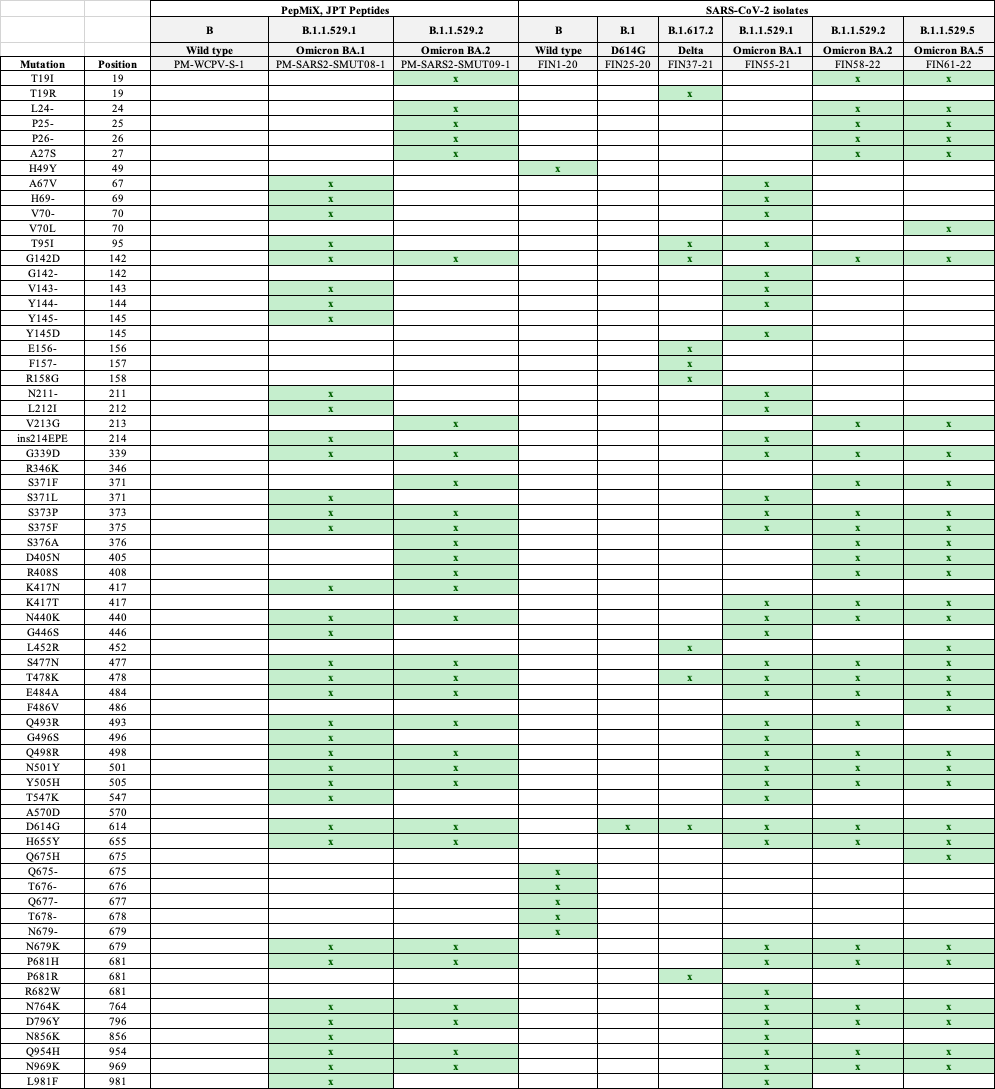


**Supplementary Table 2.** Antibodies conjugated with fluorochromes used in activation induced marker (AIM) assay.

| **Antibody** | **Fluorochrome** | **Amount used** | **Manufacturer** | **Cat#** |
| --- | --- | --- | --- | --- |
| Anti-human CD45 | APC-eFluor780 | 5 μl/test | Invitrogen/Life technologies | 47-0459-42 |
| Anti-human CD3 | eFluor506 | 5 μl/test | Invitrogen/Life technologies | 69-0038-42 |
| Anti-human CD4 | eFluor450 | 5 μl/test | Invitrogen/Life technologies | 48-0049-42 |
| Anti-human CD8a | PerCP-eFluor710 | 5 μl/test | Invitrogen/Life technologies | 46-0087-42 |
| Anti-human CD69 | PE | 15 μl/test | BD Biosciences | 555531 |
| Anti-human CD134 | PE/Cyanine7 | 5 μl/test | BioLegend | 350012 |
| Anti-human CD137 | APC | 5 μl/test | BioLegend | 309810 |
| Anti-human CD45RA (HI100 clone) | Brilliant Violet 785 | 5 μl/test | BioLegend | 304140 |
| Anti-human CD197 (CCR7) (G043H7 clone) | PE/Dazzle 594 | 5 μl/test | BioLegend | 353236 |
| Anti-human CD185 (CXCR5) (J252D4 clone) | Brilliant Violet 605 | 5 μl/test | BioLegend | 356930 |

**
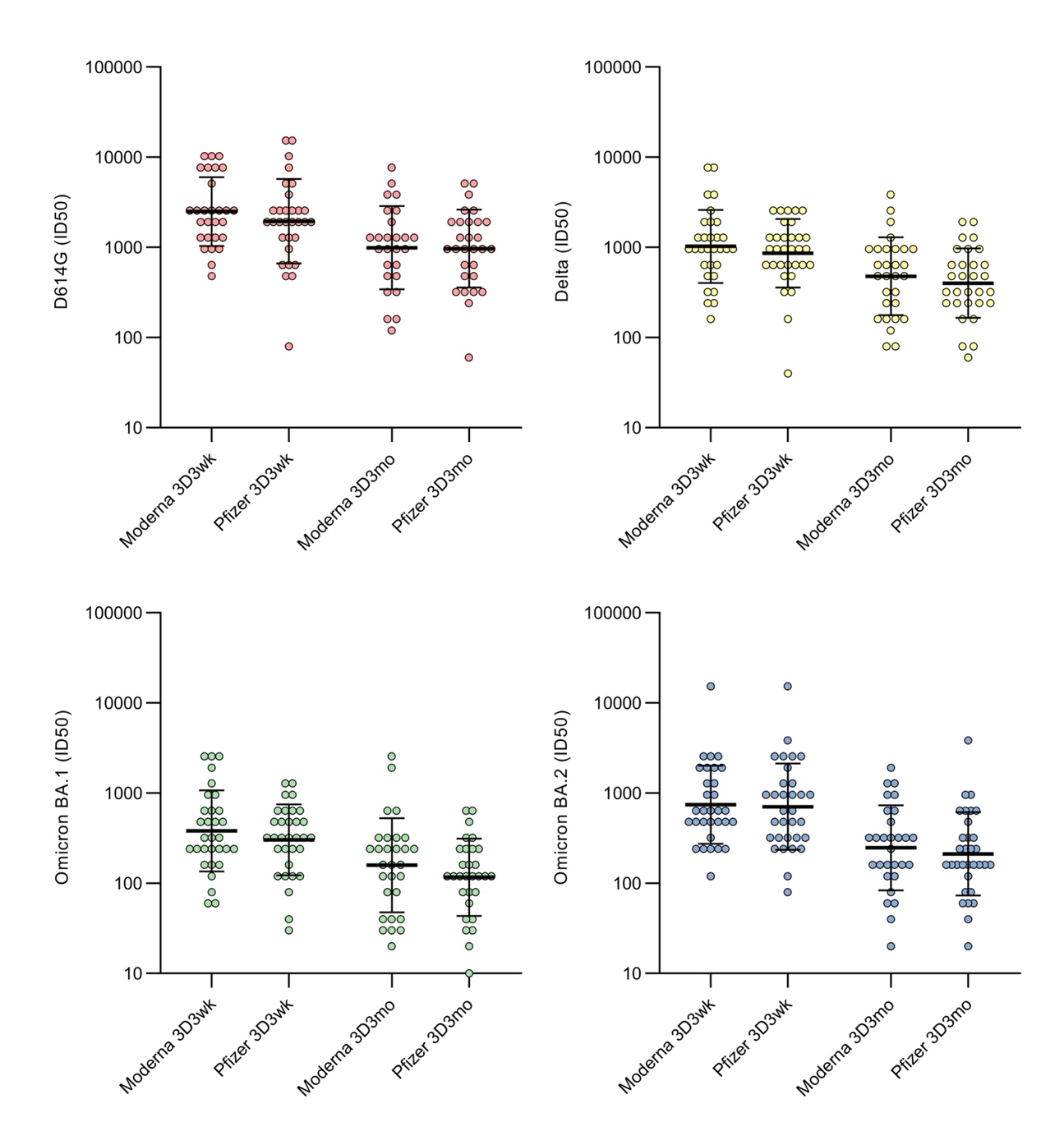
**

**Supplementary Figure 1.** Neutralization titers against D614G strain, and Delta, Omicron BA.1 and BA.2 variants after the third mRNA vaccine dose (Moderna vs Pfizer). Differences were compared with Mann Whitney U-test. There were no statistical differences between the vaccine induced antibody responses at the same time points).


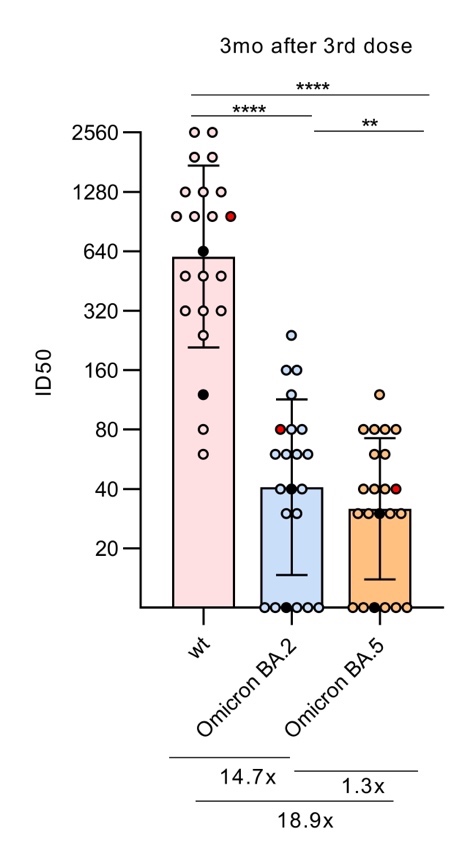


**Supplementary Figure 2.** Neutralizing antibodies against wild type (wt) strain and Omicron variants three months after the third vaccine dose. Microneutralization test was carried out in laboratory B in VeroE6 cells using wild type strain or Omicron BA.2 or BA.5 variants as described in methods. Statistical differences were compared with Mann Whitney U-test. ** p<0.01, **** p<0.0001.


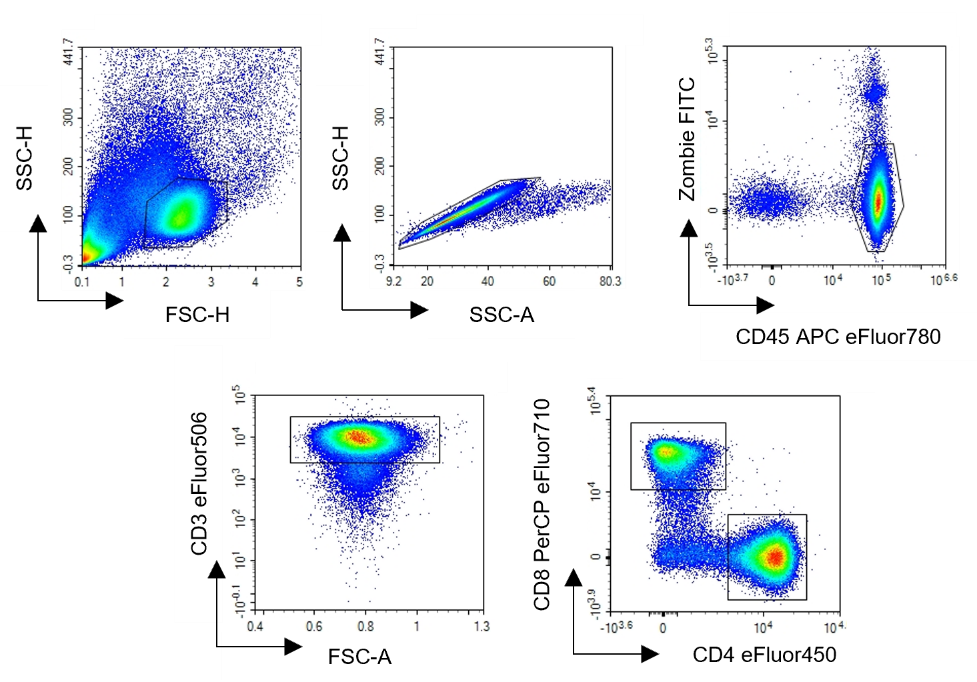


**Supplementary Figure 3.** Representative flow cytometry plots and gating strategy representing lymphocyte population subgated to singlets, live CD45^+^ cells, CD3^+^ cells, and CD4^+^ and CD8^+^ T cells.


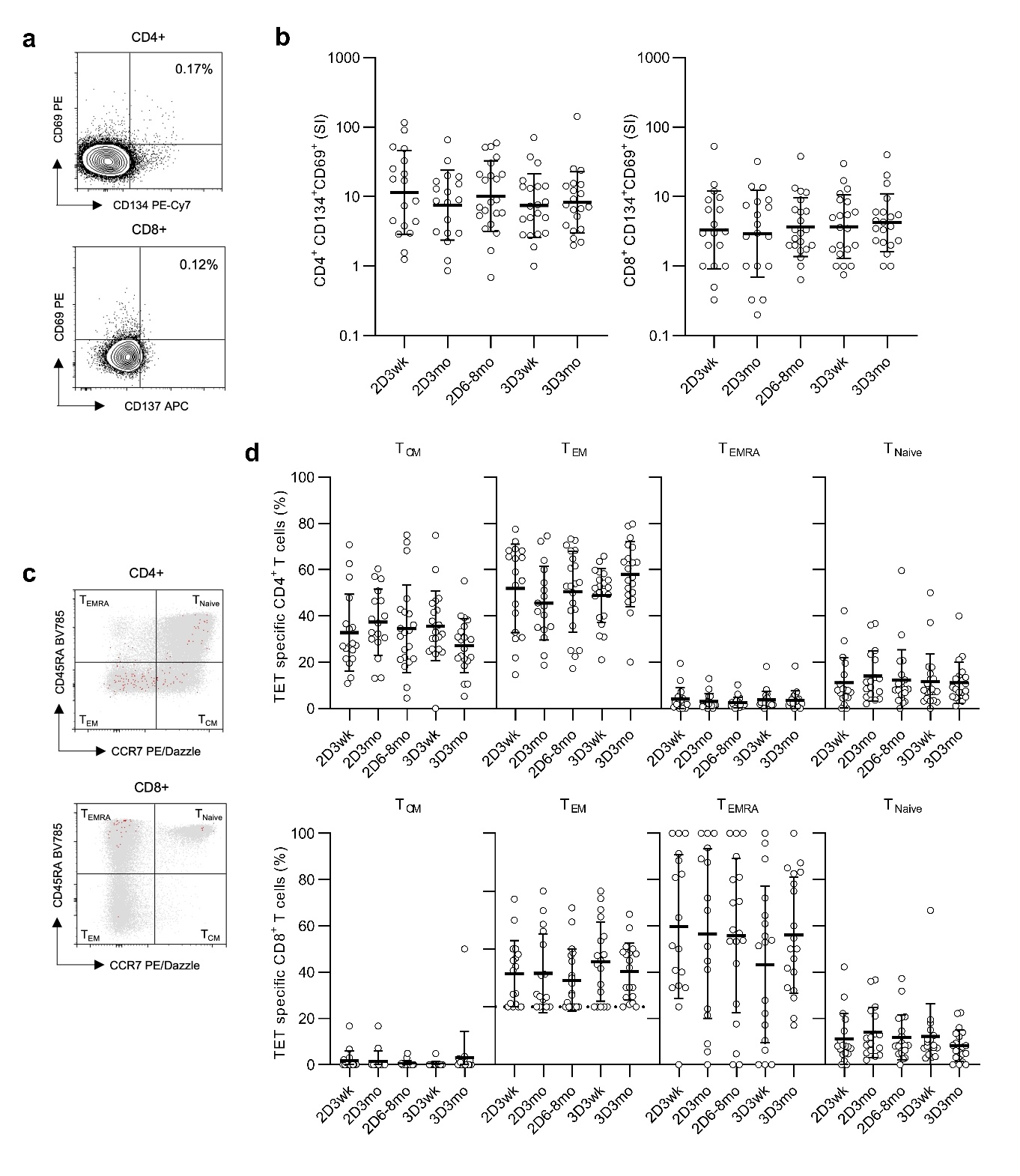


**Supplementary Figure 4. CD4^+^ and CD8^+^ T cell responses against tetanus toxoid. a,** Representative flow cytometry plots and the gating of CD4^+^ cells expressing CD69^+^CD134^+^ and CD8^+^ cells expressing CD69^+^CD137^+^ after stimulation with tetanus toxoid (TET). **b,** Longitudinal TET specific CD4^+^ and CD8^+^ T cell responses in 32 vaccinated HCWs three weeks (2D3wk), three months (2D3mo), and six to eight months (2D6–8mo) after the second vaccine dose, and three weeks (3D3wk) and three months (3D3mo) post the third vaccine dose. Data are shown as stimulation indices (SI) relative to DMSO-stimulated PBMCs. **c,** Gating and distribution of memory phenotypes of total CD4^+^ and CD8^+^ (gray) and activated CD4^+^CD69^+^CD134^+^ and CD4^+^CD69^+^CD137^+^ (red) T cells after stimulation with TET. **d,** Proportions of memory phenotypes of TET specific CD4^+^ and CD8^+^ T cells after the second and third vaccine doses.

**
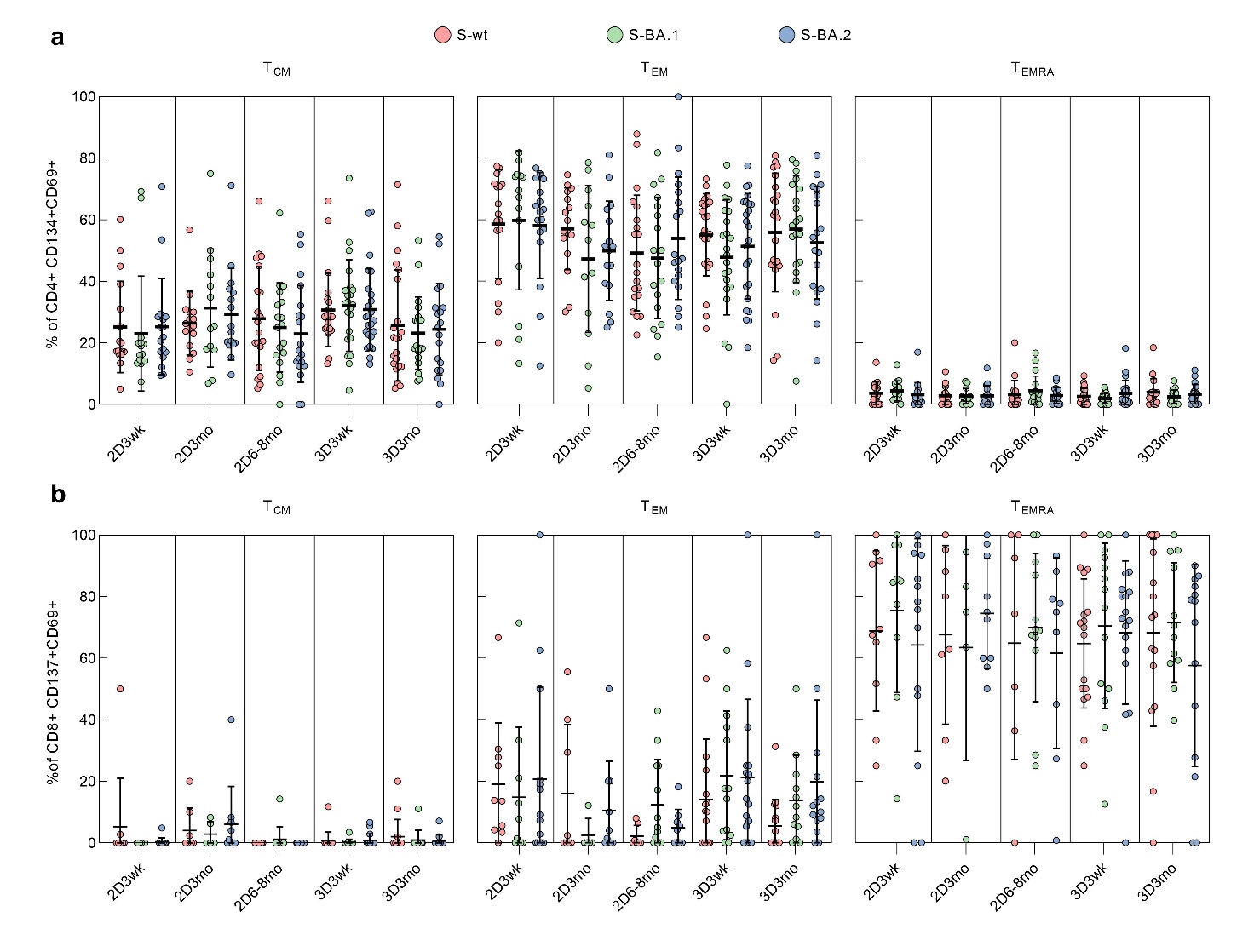
**

**Supplementary Figure 5.** Distribution of spike-specific **a**, CD4^+^ and **b**, CD8^+^ T cells into memory subsets after the second (2D) and third (3D) vaccine doses. PBMCs were stimulated with SARS-CoV-2 spike peptide pools of the wild type strain (S-wt) and Omicron BA.1 and BA.2 variants (S-BA.1 and -BA.2). CM; central memory, EM; effector memory, TEMRA; terminally differentiated T effector memory.


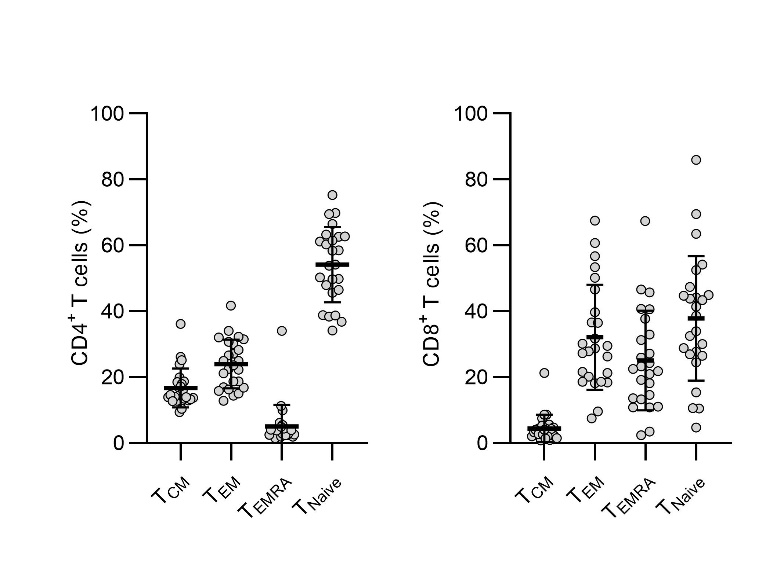


**Supplementary Figure 6.** Distribution of the total CD4^+^ and CD8^+^ T cells into memory phenotypes. Percentages are calculated as an average from S-wt stimulated PBMCs.
